# Gualou Xiebai Decoction, a Traditional Chinese Medicine, Prevents Cardiac Reperfusion Injury of Hyperlipidemia Rat via Energy Modulation

**DOI:** 10.3389/fphys.2018.00296

**Published:** 2018-04-04

**Authors:** Lu-Lu Yan, Wei-Yang Zhang, Xiao-Hong Wei, Li Yan, Chun-Shui Pan, Yang Yu, Jing-Yu Fan, Yu-Ying Liu, Hua Zhou, Jing-Yan Han, Xin-Sheng Yao

**Affiliations:** ^1^State Key Laboratory of Quality Research in Chinese Medicine, Macau University of Science and Technology, Macau, China; ^2^Tasly Microcirculation Research Center, Peking University Health Science Center, Beijing, China; ^3^Department of Integration of Chinese and Western Medicine, School of Basic Medical Sciences, Peking University, Beijing, China; ^4^Institute of Traditional Chinese Medicine and Natural Products, College of Pharmacy, Jinan University, Guangzhou, China

**Keywords:** cardiac function, myocardial structure, ATP synthase subunit, apoptosis, Rho-associated protein kinase, Rho GTPases

## Abstract

**Background:** Gualou Xiebai Decoction (GLXB) is a classic prescription of Chinese medicine used for the treatment of cardiac problems. The present study was designed to explore the effect and mechanism of GLXB on ischemia/reperfusion (I/R) induced disorders in myocardial structure and function, focusing on the regulation of energy metabolism and the RhoA/ROCK pathway.

**Methods:** After hyperlipidemic rat model was established by oral administration of high fat diet, the rats were treated with GLXB for 6 weeks and subjected to 30 min occlusion of the left anterior descending coronary artery (LADCA) followed by 90 min reperfusion to elicit I/R challenge. Myocardial infarct size was assessed by Evans blue-TTC staining. Myocardial blood flow (MBF) and cardiac function were evaluated. Enzyme-linked immunosorbent assay was performed to examine the content of ATP, ADP, AMP, CK, CK-MB, LDH, cTnT, cTnI, and IL-6. Double staining of F-actin and terminal deoxynucleotidyl transferase-mediated dUTP nick end labeling was conducted to assess myocardial apoptosis. Expressions of ATP synthase subunit δ (ATP 5D), and RhoA and ROCK were determined by Western blotting.

**Results:** Administration with GLXB at high dose for 6 weeks protected heart against I/R-induced MBF decrease, myocardial infarction and apoptosis, ameliorated I/R-caused impairment of cardiac function and myocardial structure, restored the decrease in the ratio of ADP/ATP and AMP/ATP, and the expression of ATP 5D with inhibiting the expression of RhoA and ROCK.

**Conclusions:** Treatment with GLXB effectively protects myocardial structure and function from I/R challenge, possibly via regulating energy metabolism involving inactivation of RhoA/ROCK signaling pathway.

## Introduction

Despite advancements, cardiovascular diseases are considered a leading burden for public health worldwide (Bayeva et al., 2013; Dzau, 2016), among which coronary heart disease (CHD) is the most common form. CHD is a complex condition caused by many factors. Of them, hyperlipidemia is a major risk factor for the induction and development of CHD (Balakumar and Babbar, 2012). Hyperlipidemia elevates levels of serum lipids and triglycerides, which in turn lead to heart diseases such as atherosclerosis, CHD, myocardial infarction and stroke (Gupta et al., 2015; Zhang et al., 2017).

After an acute myocardial infarction, early and successful myocardial reperfusion using thrombolytic therapy or primary percutaneous coronary intervention (PCI) is the most effective strategy for reducing the size of myocardial infarct and improving the clinical outcome. The process of restoring blood flow to the ischemic myocardium, however, can induce injury. This phenomenon known as myocardial reperfusion injury can paradoxically reduce the beneficial effects of myocardial reperfusion (Yellon and Hausenloy, 2007). The clogged arteries further exaggerate myocardial ischemic injury due to the deposition of lipids and the endogenous cellular mediators released in hyperlipidemic state (Gupta et al., 2015).

Imbalances in metabolic supply and demand within the ischemic organ lead to hypoxia and microvascular dysfunction (Eltzschig and Eckle, 2011). A key factor during insufficient perfusion of myocardial tissue is the destruction of mitochondrial oxidative phosphorylation resulting in the depletion of adenosine triphosphate (ATP). Insufficient ATP-production and resultant intracellular acidosis promote an activation of the Na^+^/H^+^ and the Na^+^/Ca^2+^ exchanger, causing intracellular sodium and calcium overload with loss of resting potential and cellular swelling. Calcium overload and increased level of reactive oxygen due to breakdown of respiratory chain provoke prolonged opening of mitochondrial permeability transition pores (MPTPs) resulting in mitochondrial swelling and rupture, cytochrome C release, and induction of cell apoptosis (Salameh et al., 2017). In addition, the role of RhoA/ROCK signaling in I/R injury has attracted increasing attention (Budzyn et al., 2006; Dong et al., 2010). Studies have shown that the I/R-decreased mitochondrial ATP synthase subunit and energy metabolism disorders can be attenuated by inhibiting the ROCK pathway (He et al., 2014; Cui et al., 2017).

I/R-induced cardiac injury involves a variety of pathological processes that change as the injury progresses. In recent years, studies have shown that traditional Chinese medicine has the advantages of multi-target treatment of I/R-induced injury (Han et al., 2017). Gualou Xiebai Decoction (GLXB), composed of *Trichosanthis Pericarpium, Allii Macrostemonis Bulbus*, and wine, is a classic prescription of the famous doctor Zhang Zhongjing in the Han Dynasty of China (AD 200-205). It has been used to deal with heart conditions in China for thousands years. Modern pharmacological research results show that GLXB has a diversified activities including protecting myocardium, anti-oxidation, anti-apoptosis, anti-inflammation, anti-hypoxia, improving blood rheology, anticoagulation, anti-fibrosis, regulation of neurotransmitters and cardiac functions, among others. *T. Pericarpium* and *A. Macrostemonis Bulbus* have also been reported to exert lipid-lowering effect. A number of studies are available regarding the effect of this medicine on I/R-induced myocardial injury in normal animals, showing the potential of GLXB in reducing the electrocardiogram ST-segment elevation induced by myocardial I/R and the range of myocardial infarction, protecting myocardial tissue, decreasing the lactic dehydrogenase (LDH) level, creatine kinase (CK-MB), and malonaldehyde (MDA) activity, increasing the activity of serum superoxide dismutase (SOD), and improving myocardial fibrosis caused by ischemia model. The proposed mechanisms involved in its effects include: inhibiting the activity of nitric oxide synthase (NOS) and reducing the excessive nitric oxide (NO); inhibiting mitogen activated protein kinase (MAPK) signaling pathway; inhibiting the expression of caspase3, and promoting the expression of phosphorylated Akt protein, and inhibiting the phosphorylation of p38 MAPK in myocardial tissue. The action on chronic myocardial ischemia has been shown via removal of oxygen free radicals, inhibition of P38, JNK, ERK1/2 protein phosphorylation. However, no study is available regarding the effect of GLXB on the I/R-induced myocardial injury in the hyperlipidemia subject, a condition more closer to the clinical practice due to the higher morbidity of myocardial I/R in people with hyperlipidemia.

The purpose of present study was to explore the effect of GLXB on I/R-induced impairment on myocardial structure and cardiac functions in hyperlipidemia rats, with focus on the possible implication of GLXB in energy metabolism modulation.

## Materials and methods

### Animals

Male Sprague–Dawley rats, 4 weeks old, weighing 95 ± 10 g, were purchased from the Animal Center of Peking University with the certificate number SCXK 2016-0010. The rats were housed in cages at temperature 22 ± 2°C, humidity 40 ± 5%, under a 12-h light/dark cycle, and received standard diet or high fat diet (HFD) and water *ad libitum*. The rats were fasted for 12 h before experiment but allowed to access water freely.

The experimental procedures were in accordance with the recommendations of guidelines, EU Directive 2010/63/EU for animal experiments. Experiment protocols were approved by Peking University Biomedical Ethics Committee Experimental Animal Ethics Branch (LA2010-001). The authors of this manuscript have certified that they comply with the Principles of Ethical Publishing in the International Journal of Cardiology.

### Herbs

The decoction pieces of *T. Pericarpium* and *A. Macrostemonis Bulbus* were purchased from Handan Chinese Herbal Medicinal Materials Company (Handan, China) and identified by Prof. Guang-Xiong Zhou of Jinan University. The quality of the decoction pieces complied with the requirements of the two herbs in Chinese Pharmacopoeia Edition 2015. Voucher specimens were deposited in the Institute of Traditional Chinese Medicine & Natural Products, Jinan University (No. JNU-TK-201503 and No. JNU-AM-201503).

### Extraction and isolation of GLXB

To prepare GLXB extract, 15 kg dry *T. Pericarpium* and 10 kg *A. Macrostemonis Bulbus* were mixed and extracted by heat-reflux in 60% ethanol, yielding 5 kg exact (GX) after removing the solvents *in vacuo*.

### UHPLC-TOF-MS analysis of GLXB extract

The total extract was first passed through a solid-phase extraction column (SPE, Phenomenex Strata™-C18E, 500 mg, 2 cc, Phenomenex, California, USA), eluted with 2 mL water and 2 mL methanol, successively. The eluted solutions were poured together and evaporated, and then re-dissolved in 50% acetonitrile at a concentration of 8.0 mg/mL. The resultant sample was subjected to chromatographic analysis on an Agilent 1290 UHPLC system (Agilent Technologies, California, USA) equipped with a Waters Acquity™ BEH-C18 column (ϕ10 × 100 mm, 1.7 μm) and the MS spectra were acquired by an Agilent 6230 HR-TOF-MS system. The mobile phases were (A) 0.1% formic acid in water (B) and 0.1% formic acid in acetonitrile, and the gradient elution program was (time/B%): 0–5 min, 3–10%; 5–15 min, 10–15%; 15–16 min, 15–18%; 16–20 min, 18–20%; 20–30 min, 20–30%; 30–35 min, 30–40%. The chromatographic analysis was performed at 40°C with a flow rate 0.4 mL/min and injection volume 4 μL. The mass spectrometer parameters were as follows: drying gas was 325°C at 8 L/min, nebulizer pressure was 35 psig, sheath gas flow was at 11 L/min, fragmentor voltage was 175 V and skimmer voltage was 65 V. Acquisition was conducted in positive mode and mass range was 50–2,000 Da. All the chemical reagents mentioned above were UPLC grade, and water was prepared with a Millipore Ultrapure System (Merck Millipore, Darmstadt, Germany).

### Other reagents

Evans blue was purchased from Sigma-Aldrich Ltd. (St. Louis, USA), freshly prepared to 4% solution with saline before experiment. 2,3,5-triphenyltetrazolium chloride (TTC) was from Sigma-Aldrich Ltd (St. Louis, USA), and prepared to 0.375% solution with phosphate buffer. Pentobarbital sodium was purchased from Beijing Chemical Agent Ltd. (Beijing, China). Triglycerides (TG), total cholesterol (TC), high-density lipoprotein cholesterol (HDL-C), low density lipoprotein cholesterol (LDL-C) detection reagents were purchased from Hitachi high-tech Co., Ltd. (Tokyo, Japan). CK, CK-MB, cTnT, cTnI, LDH, IL-6, ATP, ADP, and AMP ELISA Kits were from Shanghai BlueGene Biotech CO., Ltd (Shanghai, China). The antibody against ATP5D was from Santa Cruz (California, USA). The antibody against ROCK1 and RhoA were obtained from Abcam (Cambridge, MA, USA). Simvastatin was from Merck Sharp & Dohme Ltd. (Haarlem, the Netherlands).

### Animal model and drug administration

One hundred and fourteen rats were fed daily with high fat diet (Research Diets D12451, 45 kcal % fat) (Beijing ncobio Co., Ltd., Beijing, China) (Hambly et al., 2005; Seo et al., 2012; Lu et al., 2017; Wang et al., 2017) for 4 weeks. Blood was taken from rats through intraocular canthus penetration, centrifuged at 956 g for 10 min, then the serum was collected. The contents of TG, TC, HDL-C, and LDL-C were tested once a week until the 9th week by HITACHI7020 automatic biochemical analyzer (Hitachi high-tech Co., Ltd., Tokyo, Japan). The 4 indicators of blood lipids significantly differed from that in standard feed rats after 9 weeks, showing that hyperlipidemia rat model was established.

Hyperlipidemia rats were randomly divided into 6 groups, 19 in each, including 2 Sham-operated groups and 4 I/R-injured groups. I/R-injured rats were randomly assigned to I/R + Saline group, I/R + GLXB low dose group, I/R + GLXB high dose group and IR + Simvastatin group. Rats were administered with GLXB or Simvastatin at the dose indicated or saline by gavage daily for 6 weeks, and remained fed with high fat diet during the period of treatment. Twelve SD rats fed with standard diet were used as control (See Table 1 for detail).

**Table 1:** Experimental grouping.

**Group**	**High fat diet**	**I/R**
Control	(–)	(–)
Sham	(+)	(–)
Sham GLXB high dose	(+)	(–)
I/R	(+)	(+)
I/R + GLXB low dose	(+)	(+)
I/R + GLXB high dose	(+)	(+)
I/R + Simvastatin	(+)	(+)

*Sham group, Sham animals receiving saline at 4 ml/kg; Sham GLXB high dose, Sham animals receiving pre-treatment with GLXB 4 g/kg; I/R, I/R-challenged animals receiving saline at 4 ml/kg; I/R + GLXB low dose, I/R-challenged animals receiving pre-treatment with GLXB at 2 g/kg; I/R + GLXB high dose, I/R-challenged animals receiving pre-treatment with GLXB at 4 g/kg; I/R + Simvastatin, I/R-challenged animals receiving pre-treatment with Simvastatin at 4 mg/kg; Control group, Animals fed on standard diet receiving saline at 4 ml/kg*.

### I/R challenge and myocardial infarct size assessment

Animals were anesthetized with 2% pentobarbital sodium (60 mg/kg) by intraperitoneal injection, and placed in a supine position. A tracheal cannula was inserted via mouth, with one end being connected with an animal breathing apparatus (ALC-V8, Shanghai Alcott Biotech Co., Shanghai, China) with 1:1 respiration rate, the frequency 75/min, and tidal volume 12 ml/kg. A thoracotomy was performed to expose the heart, and the proximal left anterior descending coronary artery (LADCA) was ligated with a 5/0 silk. Post 30 min the suture silk was released for reperfusion. The animals in Sham group underwent the same treatment except for the ligation of suture silk. The LADCA was ligated again 90 m after reperfusion, and 2 ml of 0.35% Evans blue was administrated via femoral vein. Hearts were rapidly excised and sliced into 5 sections of 1 mm thick from the apex cordis to the ligation site. To reveal the infarction, the slices were incubated with a 0.375% solution of TTC at 37°C for 15 min, and then photographed with a stereomicroscope connected with Digital Sight (DS-5M-U1, Nikon, Nanjing, China). Myocardial infarct area (IA), myocardial area at risk (AAR) and left ventricle size (LV) were analyzed on each slice by Image-Pro Plus 6.0 (Media Cybernetic, Bethesda, MD, USA) (*n* = 6) (Lin et al., 2013). The average ratios of AAR/LV (%) and IA/AAR (%) from 5 slices were used to denote the degree of myocardial ischemia and infarction.

### Myocardial blood flow

After exposure of heart, myocardial blood flow (MBF) was measured by a Laser-Doppler Perfusion Imager (PeriScan PIM3 System; PERIMED, Stockholm, Sweden) equipped with a computer, at baseline, immediately after ischemia and 30, 60, and 90 min after reperfusion. A color-coded image was used to denote the magnitude of MBF with blue to red representing low to high (Li et al., 2018).

### Heart function

A biofunctional experimental system BL-420F (Chengdu Taimen Technology Ltd, Chengdu, China) was applied to assess the heart function at baseline, immediately after ischemia, and 90 min after reperfusion. The variables measured included heart rate (HR), left ventricular systolic pressure (LVSP), left ventricular diastolic pressure (LVDP), left ventricular end diastolic pressure (LVEDP), left ventricular maximum upstroke velocity (+dp/dtmax), and left ventricular maximum descent velocity (–dp/dtmax) (Li et al., 2018).

### ELISA assay

Blood was collected at 90 min after reperfusion and centrifuged at 4°C and 1,000 g for 15 min to separate serum using heparin as an anticoagulant. The supernatant was harvested, and the contents of CK, CK -MB, LDH, cTnT, cTnI, IL-6, ATP, ADP, and AMP were detected using rat ELISA Kit by microplate reader (MULTISKAN MK3; Thermo, San Jose, CA, USA).

At 90 min after reperfusion, rats under anesthesia were perfused with saline, and the hearts were removed. The left ventricle tissue was sampled 2 mm under ligature, frozen in liquid nitrogen, and stored at −80°C. The protein of the tissues was extracted with a protein extraction kit (Applygen Technologies, Beijing, China), as per the manufacture's instruction. In brief, 80–100 mg of tissue pieces was mixed with 1 ml of RIPA containing 5 μg/ml leupeptin, 5 μg/ml aprotinin, 5 μg/ml pepstatin, and 5 mM PMSF, homogenized, incubated on ice for 30 min, and centrifuged at 19,357 g, 4°C, for 10 min. The resultant supernatant served as whole protein, and the content of ATP, ADP, and AMP of myocardium was assessed with ELISA by microplate reader (MULTISKAN MK3; Thermo, San Jose, CA, USA), according to the manufacturer's instruction. ADP/ATP and AMP/ATP were calculated based on the standard curves.

### Histology and double staining of F-actin and terminal deoxynucleotidyl transferase-mediated dUTP nick end labeling

At 90 min after reperfusion, thoracotomy was performed, and heart was removed (*n* = 3), fixed in 4% paraformaldehyde (PFA) solution for 48 h for processing paraffin section. The paraffin sections (5 μm) were stained with hematoxylin eosin (HE) as routine.

The sections were doubly stained with rhodamine phalloidine (R415, Invitrogen, Carlsbad, CA, USA) for F-actin and a cell death detection kit (Roche, Basel, Switzerland) for terminal deoxynucleotidyl transferase-mediated dUTP nick end labeling (TUNEL), according to the manufacture's instruction, and the nucleus were labeled with Hoechest 33342. Sections were observed with a Laser Scanning Confocal Microscope (TCS SP5, Leica, Mannheim, Germany) with five view fields being selected at 40 × magnification of objective from the surrounding infarction areas of the left ventricle for each section. The numbers of the TUNEL-positive cells in the five fields were counted, using the average as a measure of apoptosis (Li et al., 2018).

### Western blotting assay

Myocardial tissue was harvested from the surrounding of infarct area of LV 90 min after reperfusion. The myocardial tissue was homogenized in lysis buffer containing the protease inhibitor. Equivalent amount of proteins were loaded, electrophoresis, and transferred onto the membranes, and incubated with antibodies against ATP5D (1:200), RhoA (1:1,000), ROCK1 (1:1,000), GAPDH (1:2,000), respectively, followed by incubation with corresponding secondary antibodies. The GADPH western blotting was performed for each membrane as a loading control.

### Statistical analysis

All data were expressed as mean ± SEM. Statistical analysis was carried out with GraphPad Prism 6 statistical software, and one-way analysis of variance was used, and then for *post-hoc* testing, Fisher's least-significant-difference test was used for multiple comparisons between groups. *P* < 0.05 was considered as statistically significant.

## Results

### UHPLC-TOF-MS chromatograph and chemical profile of GLXB

**Figure 1:**
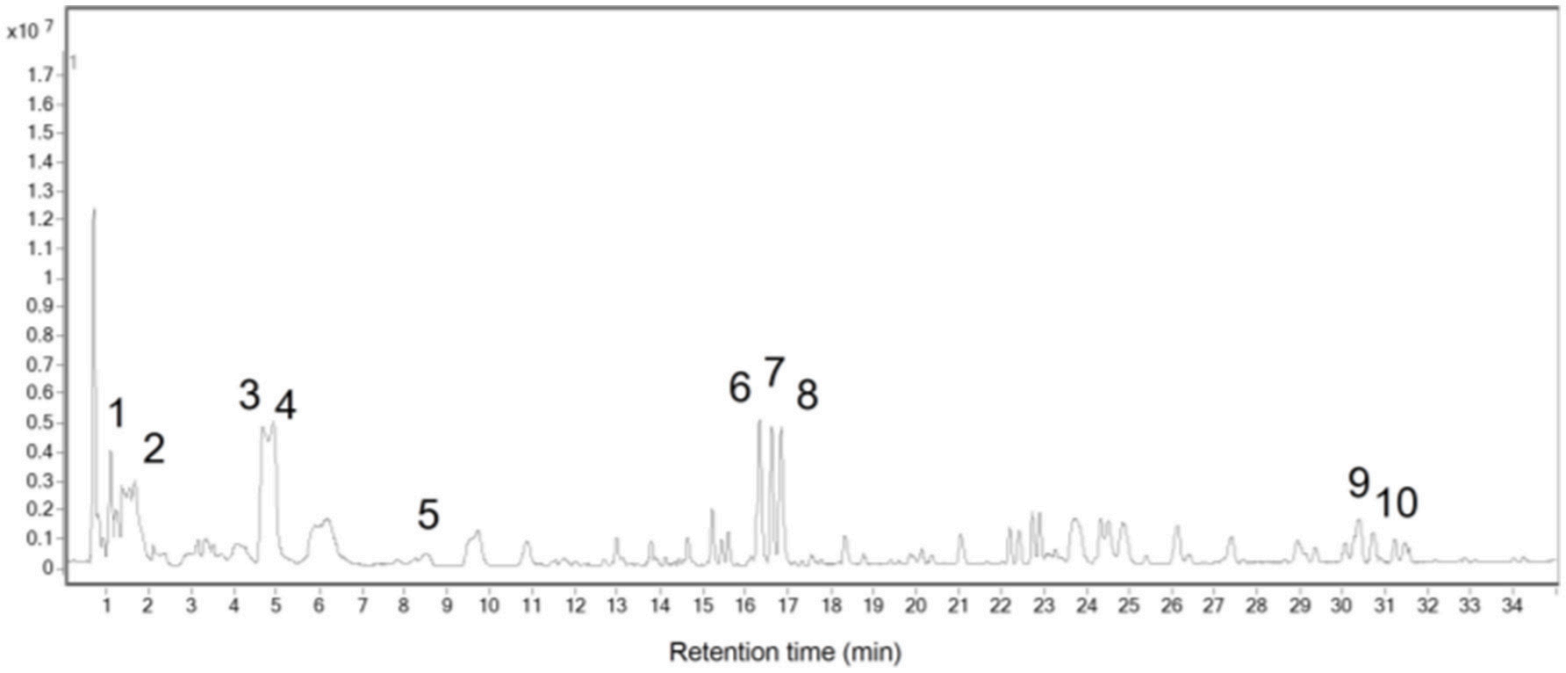
Base peaks in chromatography (BPC) of the total extract of GLXB in positive mode (time/B%): 0–5 min, 3–10%; 5–15 min, 10–15%; 15–16 min, 15–18%; 16–20 min, 18–20%; 20–30 min, 20–30%; 30–35 min, 30–40%.

To identify the chemicals contained in GLXB, UHPLC-TOF-MS Chromatography was applied, as shown in Figure 1. A total of 10 major compounds were identified by analyzing MS peaks and comparing the retention times with standard compounds, as listed in Table 2.

**Table 2:** Major compounds in the total extract of GLXB.

**No**.	***t*_R_**	**Quasi-molecular ion peak ([M+H]^+^ or [M+Na]^+^) (*m/z*)**	**Compound**
1	1.11	268.1043	Adenosine
2	1.68	268.1032	9-arabinosyladenine
3	4.86	217.0977	2,3,4,9-tetrahydro-1H-pyrido[3,4-b]indole-3*R* carboxylic acid
4	5.09	217.0978	2,3,4,9-tetrahydro-1H-pyrido[3,4-b]indole-3*S* carboxylic acid
5	8.53	169.0458	Vanillic acid
6	16.25	975.4772	Macrostemonoside Q
7	16.60	665.3488	Macrostemonoside N
8	16.82	667.3649	Macrostemonoside M
9	30.85	301.0687	Chrysoeriol
10	31.51	777.3993	Macrostemonoside S

### GLXB decreases serum TC, TG, and LDL-C, but has no influence on HDL-C

**Figure 2:**
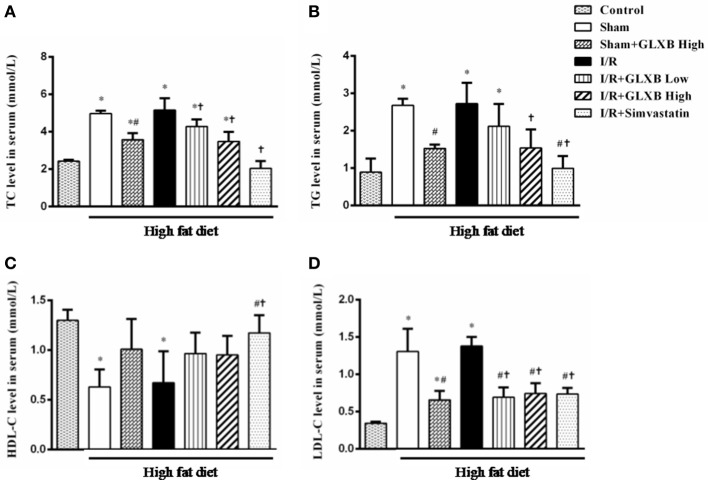
The effect of GLXB on the level of serum TC, TG, HDL-C, and LDL-C in rat. **(A)** The level of serum TC in different group. **(B)** The level of serum TG in different group. **(C)** The level of serum HDL-C in different group. **(D)** The level of serum LDL-C in different group. Data are mean ± SEM, *n* = 6. ^*^P < 0.05 vs. Control group, ^#^*P* < 0.05 vs. Sham group, ^†^*P* < 0.05 vs. I/R group.

Serum levels of TC, TG, HDL-C, and LDL-C were tested by ELISA assay to determine whether GLXB has a lipid-lowering effect. The results of serum TC, TG, HDL-C, and LDL-C are illustrated in Figure 2. Compared with Control group, the level of TC, TG, and LDL-C were significantly increased after HFD treatment, and the HDL-C level was markedly decreased. I/R insult had no effect on the level of serum TC, TG, HDL-C, and LDL-C. Interestingly, the levels of serum TC, TG and LDL-C were significantly decreased in Sham + GLXB High group, compared with both Sham group and I/R group, while the HDL-C level did not vary significantly among the four groups. GLXB at low dose exhibited similar but less effect as high dose. These results indicate the lipid-lowering activity of GLXB. Simvastatin was found able to attenuate all the four alterations induced by HFD.

### GLXB reduces I/R-induced myocardial infarct size

**Figure 3:**
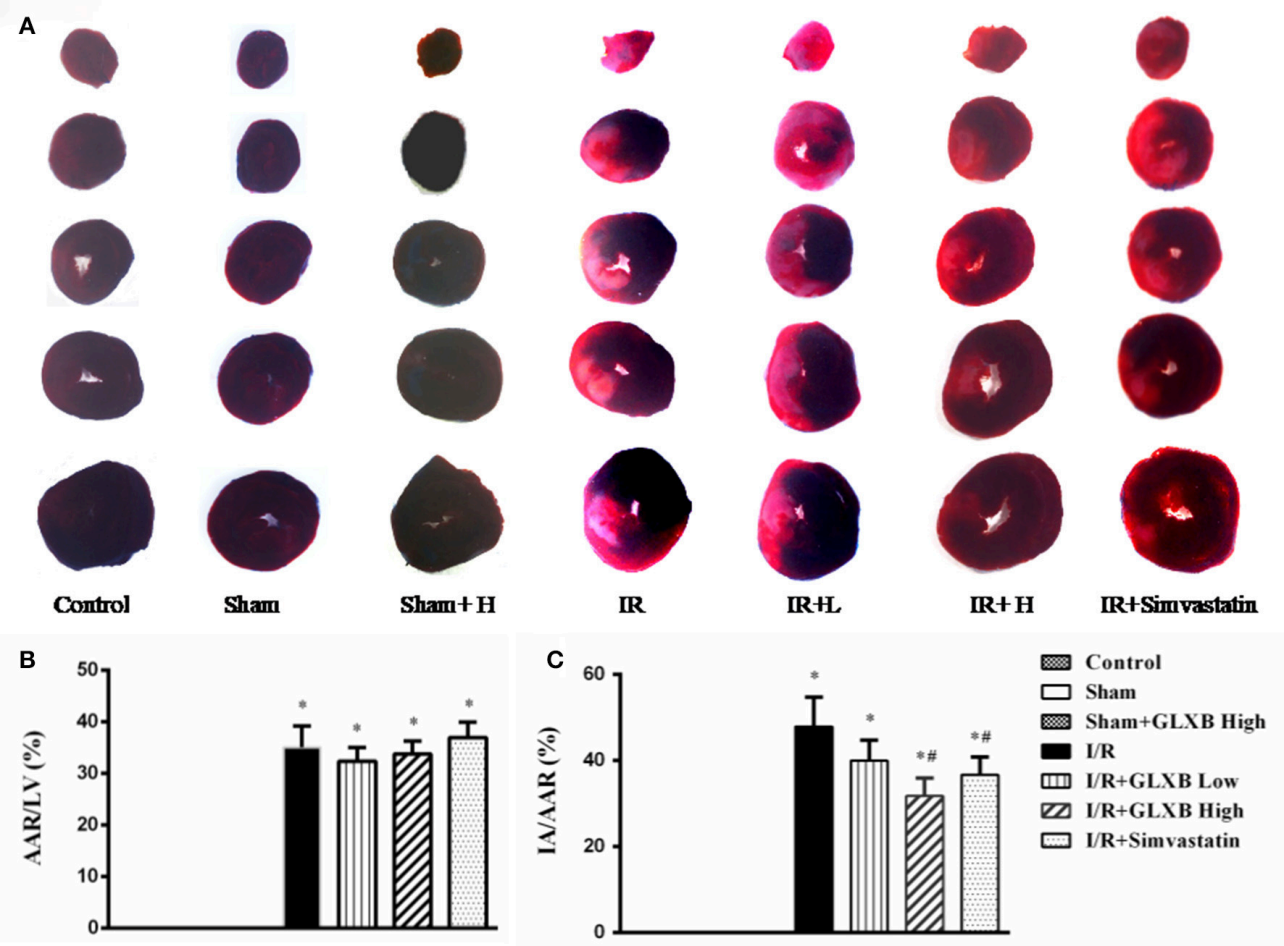
The effect of GLXB on I/R-induced myocardial infarct size in rats. **(A)** Representative myocardial tissue slices of left ventricle stained by Evans blue-TTC in Control, Sham, Sham + GLXB High, I/R, I/R + GLXB Low, I/R + GLXB High, and I/R + Simvastatin. **(B)** Quantitative analysis of AAR/LV in various groups. **(C)** Quantitative analysis of IA/AAR in various groups. Data are mean ± SEM, *n* = 6. ^*^P < 0.05 vs. Sham group, ^#^*P* < 0.05 vs. I/R group.

As a major outcome for evaluating the effect of GLXB, myocardial infarct was assessed by Evans blue-TTC staining 90 min after reperfusion and the representative images in different groups are shown in Figure 3A, wherein the pink tissue represents ischemic area, while the white tissue represents the infarction region. Apparently, myocardial tissue slices from Control group, Sham group and Sham + GLXB group exhibited no ischemia and infarct. In contrast, noticeable ischemia and infarct areas were observed in I/R groups. However, myocardial infarct areas in I/R + GLXB High group and I/R + Simvastatin group were significantly smaller than that in I/R group.

The quantitative analysis of AAR/LV and IA/AAR is shown in Figures 3B,C, respectively. Compared with Sham group, AAR/LV increased markedly in I/R group, which was not attenuated by GLXB at the two doses, nor by Simvastatin. IA/AAR in I/R group increased noticeably compared to Sham group, either, however, which was significantly alleviated by treatment with high dose of GLXB and Simvastatin. The results showed that GLXB at high dose may improve the outcome of animals underwent myocardial I/R challenge with an effectiveness similar to Simvastatin.

### GLXB attenuates I/R-induced myocardial injury

**Figure 4:**
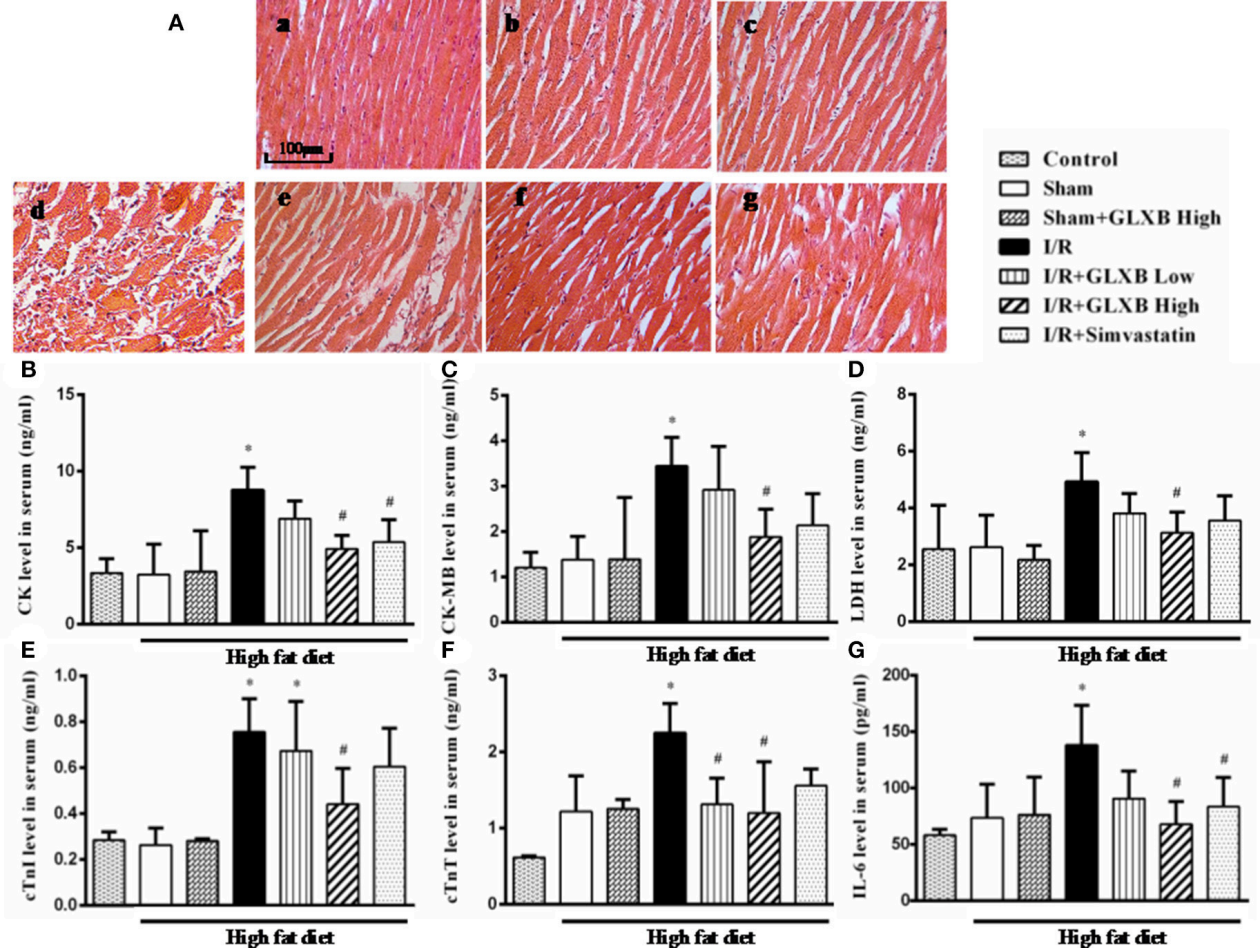
The effect of GLXB on I/R-evoked myocardial injury in rats. **(A)** Representative photographs of HE-stained myocardium (20×) in Control **(a)**, Sham **(b)**, Sham + GLXB High **(c)**, I/R **(d)**, I/R + GLXB Low **(e)**, I/R + GLXB High **(f)**, I/R + Simvastatin **(g)**. Bar = 100 μm. **(B)** Serum levels of CK in different groups. **(C)** Serum levels of CK-MB in different groups. **(D)** Serum levels of LDH in different groups. **(E)** Serum levels of cTnI in different groups. **(F)** Serum levels of cTnT in different groups. **(G)** Serum levels of IL-6 in different groups. Data are mean ± SEM, *n* = 6. ^*^P < 0.05 vs. Sham group, ^#^*P* < 0.05 vs. I/R group.

The potential of GLXB to protect myocardial tissue from I/R injury was verified further by HE staining and assessment of myocardial enzymes and inflammatory cytokines. The representative HE staining paraffin sections from different groups are shown in Figure 4A. Compared with Control group (a), the myocardium in Sham group (b) exhibited apparent cardiomyocyte hypertrophy and enlarged interstitial space, consistent with the results from others (Geetha et al., 2015). A similar pathohistology pattern was observed in Sham + GLXB High (c) group, implying no effect of GLXB on high fat diet-induced cardiomyocyte hypertrophy. Nevertheless, distinct alterations occurred in the surrounding infarction areas of myocardial tissues from I/R group (d), manifesting myocardial fibers disruption, myocardial interstitial edema, and neutrophils infiltration. Of notice, all of the I/R-evoked injuries were ameliorated by GLXB treatment, especially at the high dose of 4 g/kg (f), as well as by Simvastatin (g) but to a less extent.

Furthermore, the markers of myocardial damage were also assessed. As shown in Figures 4B–G, the serum levels of CK (Figure 4B), CK-MB (Figure 4C), LDH (Figure 4D), cTnI (Figure 4E), cTnT (Figure 4F), and IL-6 (Figure 4G) were all significantly upregulated by I/R insult, however, which were inhibited by high dose of GLXB. Low dose of GLXB and Simvastatin showed a similar trend but only a significant effect on a few markers.

These results confirm the beneficial role of GLXB at high dose in attenuating myocardial I/R injury, exhibiting an even better effect than Simvastatin did.

### GLXB attenuates I/R-evoked cardiac energy metabolism disorder and ATP 5D down regulation

**Figure 5:**
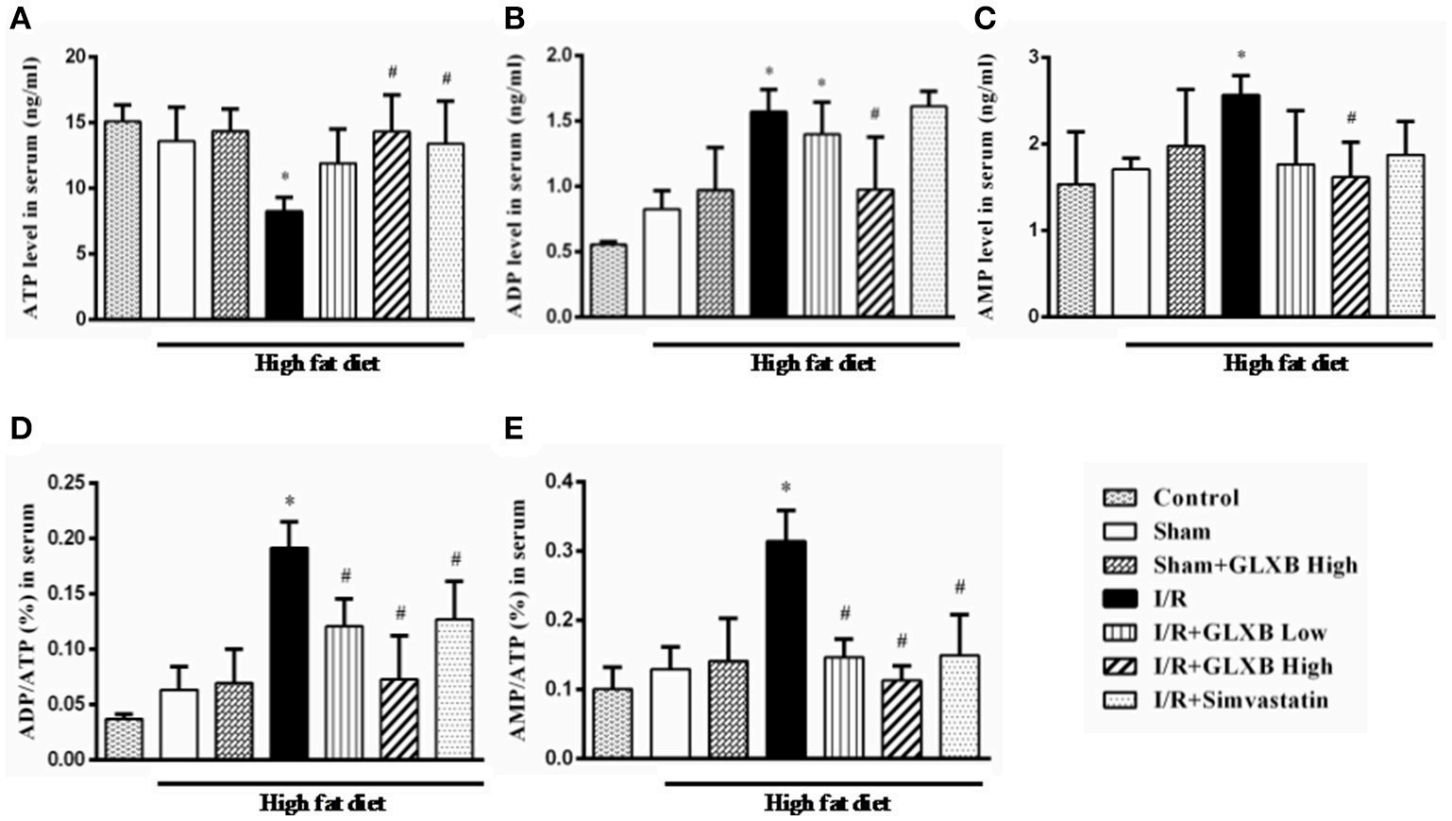
The effect of GLXB on serum level of AMP, ADP, and ATP in rats. **(A)** The serum levels of ATP in different groups. **(B)** The serum levels of ADP in different groups. **(C)** The serum levels of AMP in different groups. **(D)** The ratio of ADP/ATP in serum in different groups. **(E)** The ratio of AMP/ATP in serum in different groups. Data are mean ± SEM, *n* = 6. ^*^P < 0.05 vs. Sham group, ^#^*P* < 0.05 vs. I/R group.

**Figure 6:**
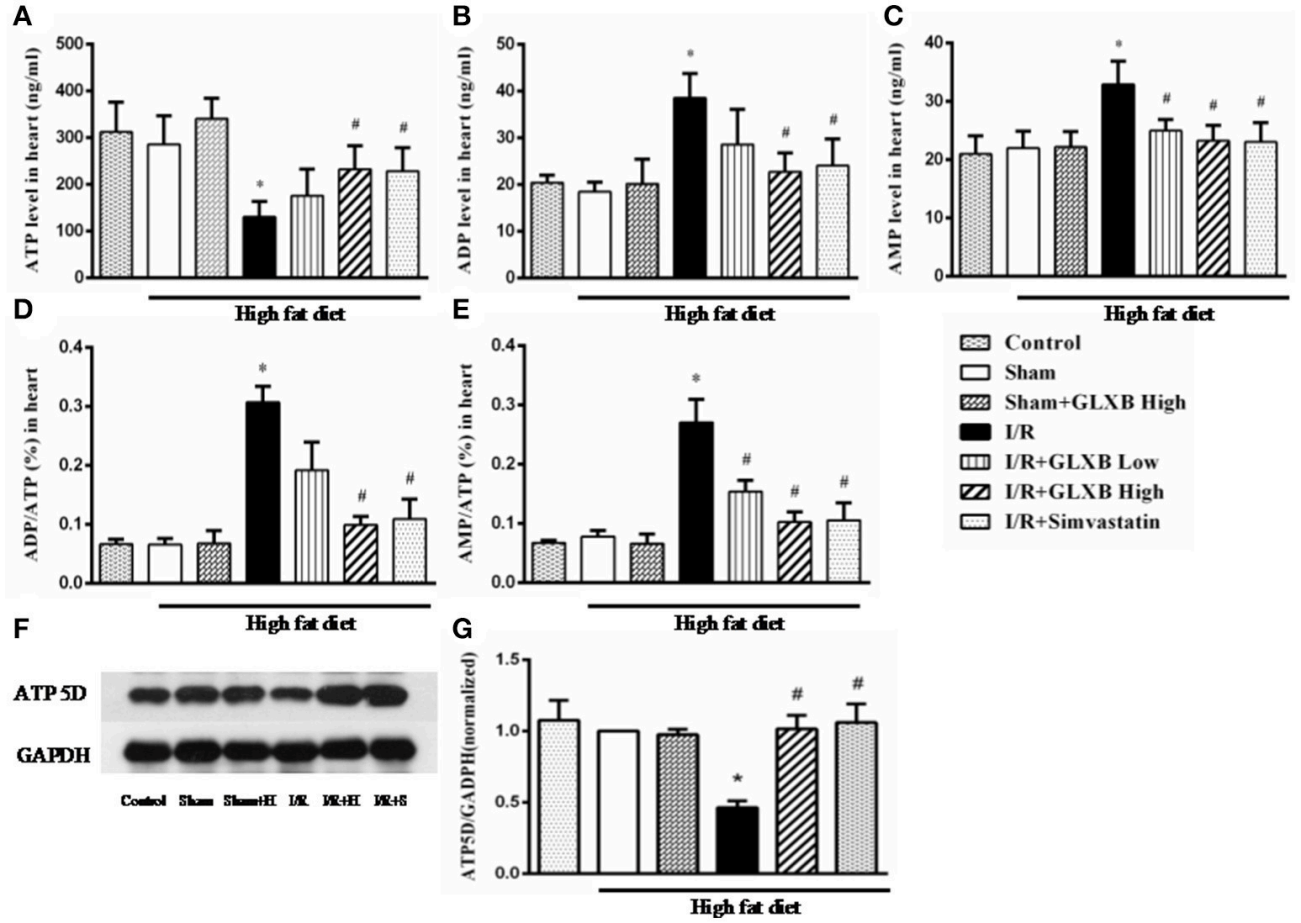
The effect of GLXB on the level of AMP, ADP and ATP in myocardial tissue of rats. **(A)** The content of ATP in myocardial tissues in different groups. **(B)** The content of ADP in myocardial tissues in different groups. **(C)** The content of AMP in myocardial tissues in different groups. **(D)** The ratio of ADP/ATP in myocardial tissues in different groups. **(E)** The ratio of AMP/ATP in myocardial tissues in different groups. Data are mean ± SEM, *n* = 6. ^*^P < 0.05 vs. Sham group, ^#^*P* < 0.05 vs. I/R group. **(F)** Representative Western blotting bands of ATP 5D in various groups. **(G)** The semi-quantitative analysis of ATP 5D in various groups. Data are mean ± SEM, *n* = 6. ^*^P < 0.05 vs. Sham group, ^#^*P* < 0.05 vs. I/R group.

Energy metabolism disorder is a key event in initiation and progression of myocardial I/R injury. Thus, experiments were undertaken to address the energy metabolism status in different conditions, in terms of the contents of ATP, ADP, and AMP in serum and myocardium. As shown in Figures 5, 6, no significant difference was observed in the three energy related variables tested between Control and Sham group, suggesting that high fat diet did not influence the energy status in rat. Noticeably, the content of ATP in both serum (Figure 5A) and myocardial tissue (Figure 6A) decreased significantly 90 min after reperfusion, as compared with Sham group, which was evidently improved by treatment of GLXB at the high dose and Simvastatin. The changes in ADP and AMP level in both serum and myocardium exhibited a trend opposite to ATP (Figures 5B–E, 6B–E), indicating a more catabolism of ATP.

As a subunit of ATP synthase, the expression of ATP 5D protein was determined by Western blotting. The representative blots from different groups and quantification of the results are displayed in Figures 6F,G and Figure S1, respectively. As noticed, the expression of ATP 5D reduced dramatically after I/R, as compared to Sham group. GLXB at the high dose and Simvastatin significantly restrained the decline of ATP 5D expression evoked by I/R.

These results demonstrate the ability of GLXB to attenuate I/R-elicited energy metabolism disorder in myocardium tissue by upregulation of ATP 5D expression, highlighting regulation of energy metabolism as a key player in the mechanism for the beneficial role of GLXB.

### GLXB attenuates RhoA/ROCK activation induced by I/R

**Figure 7:**
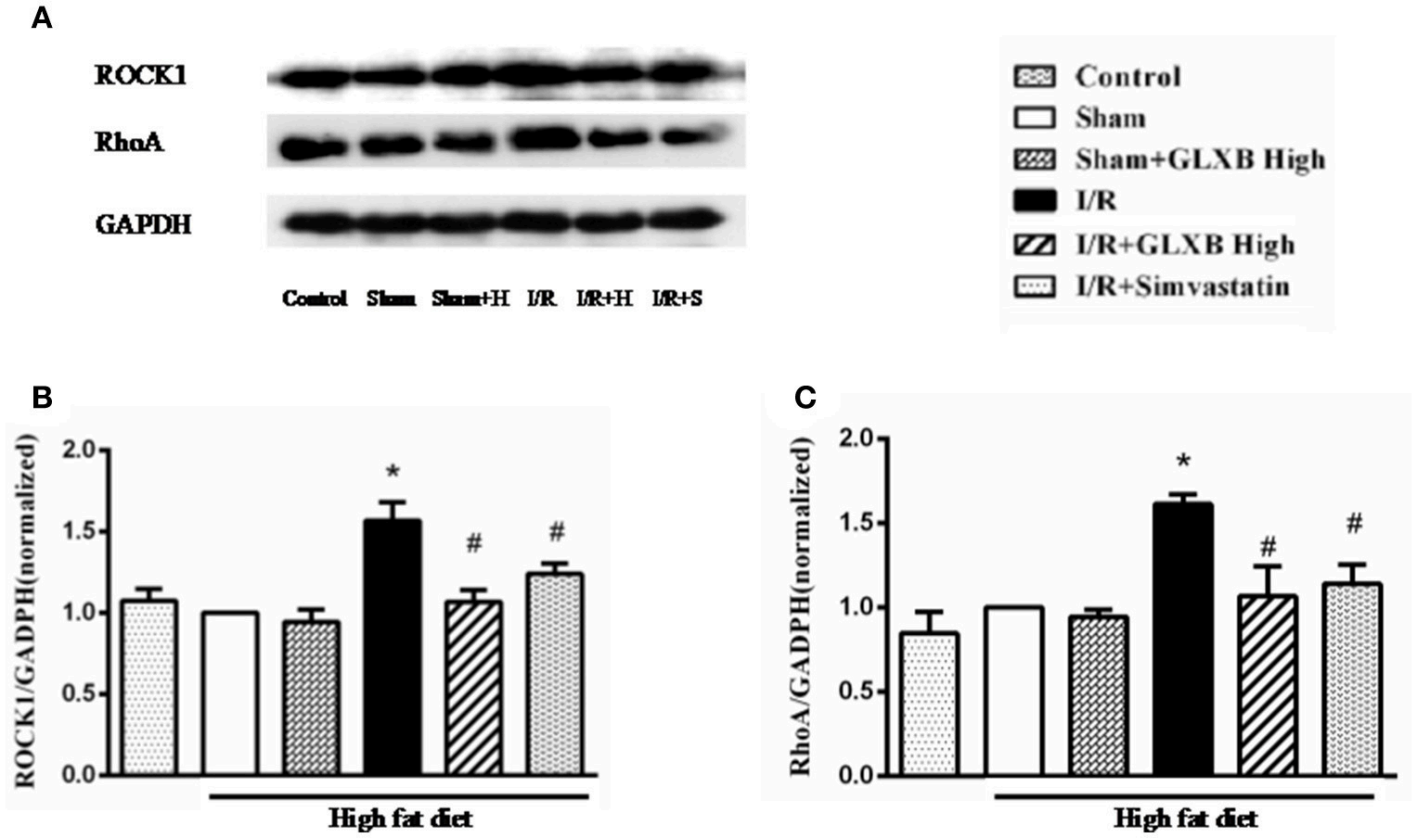
Effect of GLXB on the expression of RhoA/ROCK signaling pathway in the myocardial tissue of rats subjected to I/R. **(A)** Representative Western blotting bands of RhoA and ROCK1 in various groups. **(B)** The semi-quantitative analysis of RhoA in various groups. **(C)** The semi-quantitative analysis of ROCK1 in various groups. Data are mean ± SEM, *n* = 6. ^*^P < 0.05 vs. Sham group, ^#^*P* < 0.05 vs. I/R group.

RhoA/ROCK is a signaling pathway that participates in transcription regulation of a wide range of proteins, including ATP 5D. We thus examined RhoA/ROCK activity in different conditions to explore the likely implication of RhoA/ROCK in GLXB action. The expression of proteins implicated in RhoA/ROCK signaling pathway was assessed by using Western blotting. The results showed a significant increase in the expression of RhoA and ROCK1 in I/R-elicited myocardial tissue compared with Sham group, which was noticeably inhibited by GLXB or Simvastatin treatment (Figures 7A–C and Figure S2). This result highly suggests the involvement of RhoA/ROCK signaling pathway in the effect of GLXB, possibly via regulation of ATP5D expression thus improving energy metabolism.

### GLXB inhibits I/R-induced myocardial apoptosis

**Figure 8:**
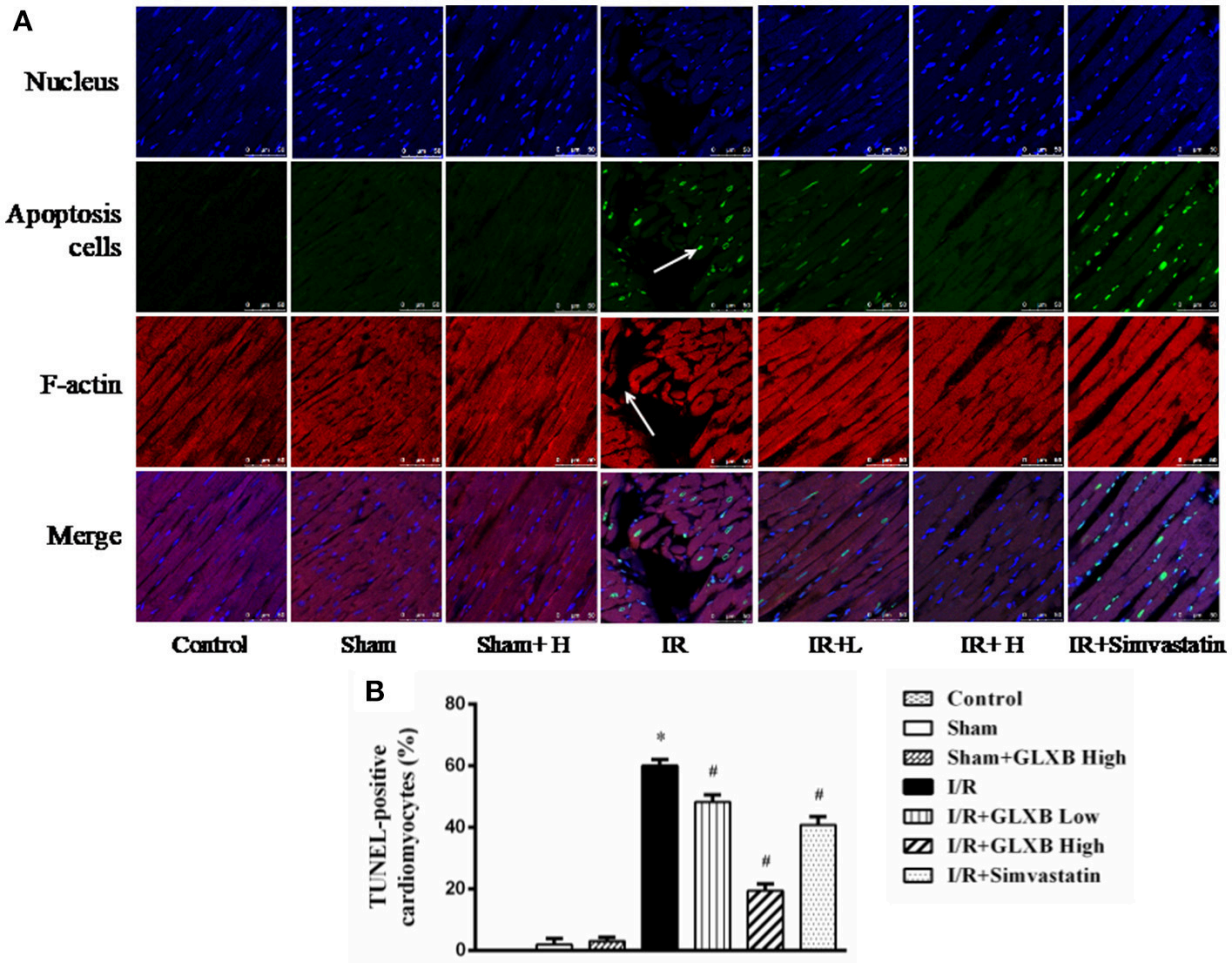
The effect of GLXB on myocardial apoptosis. **(A)** Representative photographs of double staining of TUNEL and F-actin (40×). Nucleus are stained with blue, F-actin red, and TUNEL-positive cells green. Bar = 50 μm. **(B)** Quantitative analysis of apoptosis cells in the various groups. Data are mean ± SEM, *n* = 6. ^*^P < 0.05 vs. Sham group, ^#^*P* < 0.05 vs. I/R group.

Energy metabolism disorder results in a spectrum of sequels, including cardiomyocyte apoptosis and F-actin disintegrity, among others. We next determined cardiomyocyte apoptosis and F-actin by double staining with TUNEL and rhodamine phalloidine. Figure 8 shows the representative images of various groups, wherein nuclei were stained blue, F-actin labeled by rhodamine phalloidin red, and TUNEL-positive cells green. In Control and Sham groups, no or few TUNEL-positive cells were seen. By contrast, a large number of TUNEL-positive cells were observed in I/R groups, however, which were reduced apparently by high dose of GLXB, but not by low dose of GLXB and Simvastatin. Apparently, the I/R-induced cardiomyocyte apoptosis, F-actin decrease and myocardial fibers rupture were protected by GLXB. This result is in agreement with what expected from the role of GLXB in regulation of energy metabolism.

### GLXB attenuates I/R-induced heart dysfunction

**Figure 9:**
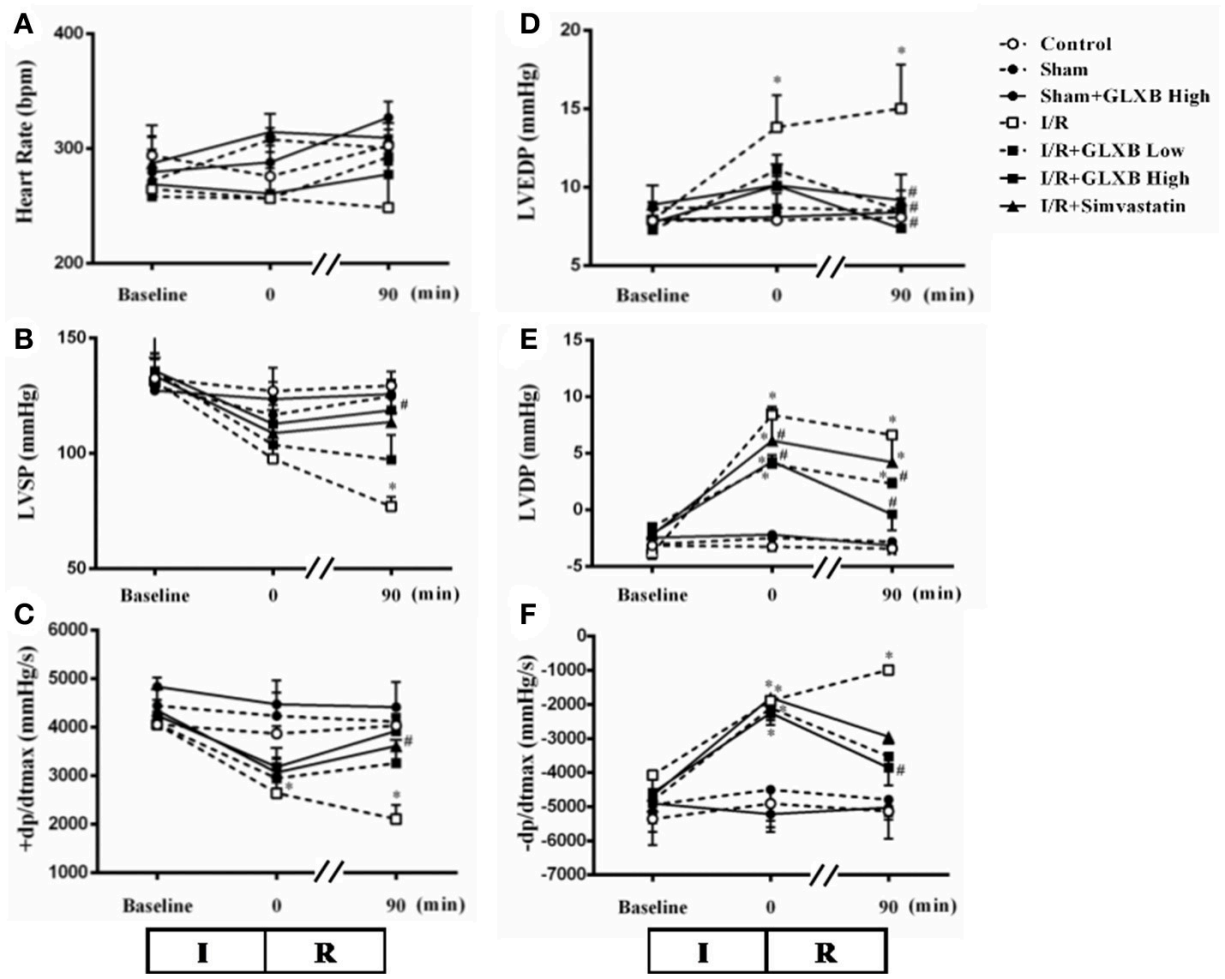
The effect of GLXB on cardiac function in rats. Presented are the time courses of HR **(A)**, LVSP **(B)**, +dp/dtmax **(C)**, LVEDP **(D)**, LVDP **(E)**, and -dp/dtmax **(F)** in various groups, respectively. The linear mixed effects models were analyzed for repeated measurement data, and least squares means were calculated between the groups of different time points. Data are mean ± SEM, *n* = 6. ^*^P < 0.05 vs. Sham group, ^#^*P* < 0.05 vs. I/R group.

Heart function is a critical target for evaluating the benefit of GLXB, which was thus assessed in different conditions at baseline, immediately after ischemia, and 90 min after reperfusion. As noticed in Figure 9, no significant difference was observed in all the 6 variables tested between Control and Sham group, suggesting that high fat diet did not affect heart function in the present setting. However, compared with Sham group, I/R caused a significant decline in LVSP and +dp/dtmax, and an apparent elevation in LVDP, LVEDP, and -dp/dtmax, indicating an impairment of heart function. Evidently, these impairments were ameliorated by pre-treatment of GLXB, especially at the dose of 4 g/kg. Simvastatin treatment revealed a protective effect similar to GLXB, but to a less extent. This result shows that the beneficial effect of GLXB on myocardial injury finally leads to an improved heart function.

### GLXB attenuates I/R-induced decrease of myocardial blood flow

**Figure 10:**
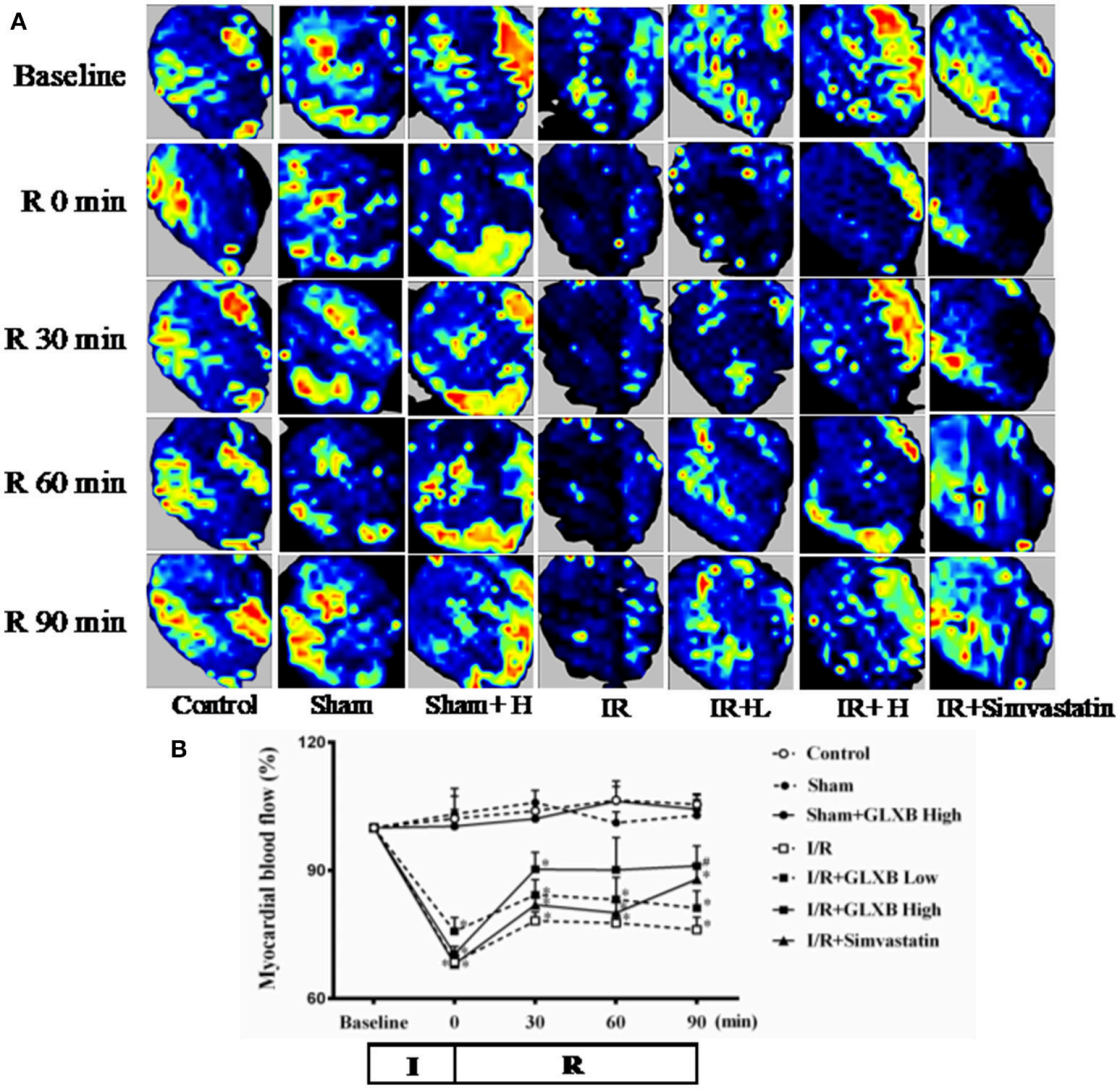
The effect of GLXB on MBF after I/R in rats. **(A)** Color images of MBF acquired by Laser Scanning Doppler Perfusion Imager in Control, Sham, Sham + GLXB High, I/R, I/R + GLXB Low, I/R + GLXB High, I/R + Simvastatin groups at Baseline, 0, 30, 60, and 90 min after reperfusion. **(B)** Time courses of MBF of rats subjected to I/R in various groups. The linear mixed effects models were analyzed for repeated measurement data, and least squares means were calculated between the groups of different time points. Data are mean ± SEM, *n* = 6. ^*^P < 0.05 vs. Sham group, ^#^*P* < 0.05 vs. I/R group.

I/R causes a decrease in MBF and an effective strategy for myocardial I/R injury should improve MBF as well. MBF was thus detected in different conditions. The color images acquired by the Laser Scanning Doppler in various groups are showed in Figure 10A. No obvious difference in MBF at baseline was observed among the seven groups, nor among Control, Sham, and Sham + GLXB High group at each time point, either. A prominent decrease in MBF occurred in I/R group immediately after ischemia, and persisted till the end of the observation. Pre-treatment with GLXB prevented MBF from decrease by I/R, with the higher dose being more efficient than lower dose. Simvastatin treatment attenuated MBF as well, but only at 90 min after reperfusion. Figure 10B is the time courses of MBF changes in the various groups at different time point, which confirmed the impression from Figure 10A. This result is parallel with the attenuating effect of GLXB on myocardial I/R injury.

## Discussion

The role of hyperlipidemia in cardiovascular diseases has long been recognized (Ferdinandy, 2003; Thakker et al., 2008), which not only presents as an independent risk factor for myocardial infarction, but also increases the severity of myocardial ischemia and reperfusion injury (Zhao et al., 2009), as evidenced by the upregulated expression of TNF-α and IL-1β in the heart (Han et al., 2016), increased left ventricular LDH, CK-MB, and collagen content (Ferdinandy et al., 1998; Giricz et al., 2006), enhanced superoxide production (Ohara et al., 1995), decreased myocardial NO concentration (Hoshida et al., 1996), excessive generation of ROS (Onody et al., 2003; Giricz et al., 2006; Csont et al., 2007), enhanced activation of apoptotic caspase-3 (Wang et al., 2002), impaired activation of the mitochondrial ATP-activated potassium channel (Katakam et al., 2007), and inhibition of MMP-2 (Giricz et al., 2006). Therefore, it is appealing to develop a strategy effective to attenuate the myocardial ischemia and reperfusion injury in the patients with hyperlipidemia.

Accumulating study has confirmed the ability of GLXB to lower the lipid in hyperlipidemia animals and attenuate the I/R-induced myocardial damage in rats with normal blood lipid. However, no study is published so far as to its effect on I/R induced myocardial injury in the hyperlipidemia subject, a condition that is more frequently encountered in clinic practice. The result of present study revealed that GLXB treatment significantly protected the myocardium from I/R injury in hyperlipidemia rat, as shown by the decreased level of CK, CK-MB, LDH, cTnI, cTnT, and IL-6, improved cardiac function, and mitigated myocardium damage, consistent with findings in animals with normal blood lipid. These results demonstrated that GLXB is able to reduce myocardial I/R injury in hyperlipidemic animals, with an efficiency similar to that for normal blood lipid animals.

The present study found that GLXB inhibits the elevation of LVEDP and LVDP, the decrease of +dp/dtmax and the increase of -dp/dtmax after I/R to improve cardiac function. The result is similar to the previous research in normal animals. On the other hand, we did not find any change in heart rate in rats after myocardial injury by I/R challenge, nor effect of GLXB on the heart rate either, which is inconsistent with the results by Ding and colleagues reporting that a deceased heart rate was observed in rats with myocardial infarction caused by ligation and subsequent release of the LADCA, while pretreatment with GLXB for 4 weeks prevented the decrease in the heart rate (Ding et al., 2016). A possible reason for this difference may be that arrhythmia occurs mostly in the early stage of reperfusion (Sun et al., 2014), while our study detected the heart rate at 90 min after reperfusion, missing the time window for finding the effect of GLXB on heart rate.

Most study regarding the mechanism for the protection of GLXB on I/R-induced myocardial infarction has focused on its effect on apoptosis, oxidative stress and inflammation, and the signaling pathways proposed include NF-κB, TGF-β1, and MAPKs (Ding et al., 2013, 2016). The new finding of the present study is that GLXB increases ATP content by up-regulating the expression of ATP 5D of ATP synthase in the myocardium, suggesting that GLXB may improve energy metabolism. Energy metabolism is well-known to be dysregulated in myocardium after ischemia-reperfusion due to the blockage of oxygen and nutrition, which leads to the depletion of ATP and initiates a spectrum of consequence, such as myocardial tissue F-actin depolymerization and myocardial tissue injury, vascular endothelial cells impairment and microcirculation dysfunction. Besides, as the major source of ATP, mitochondria disorder including the downregulation of ATP 5D may promote the production of peroxides and oxidative stress and mitochondria-mediated apoptosis, which together contribute to the decreased cardiac function (Lin et al., 2013). Our results show that GLXB can improve energy metabolism, prevent depolymerization of F-actin, attenuate myocardial tissue injury, apoptosis and cardiac dysfunction, suggesting improving energy metabolism as one of the mechanisms responsible for the protection of GLXB against I/R-injured myocardium.

The critical role of RhoA/ROCK in I/R injury has been well-recognized (Bao et al., 2004; Hamid et al., 2007; Shibata et al., 2008; Zhang et al., 2009), which is shown to inhibit PI3K/AKT/eNOS pathway, the expression of the anti-apoptotic protein Bcl-2 protein, and activation of the c-Jun NH2 terminal kinase. Particularly, ROCK inhibitors were found to increase the expression of lactate dehydrogenase and glyceraldehyde-3-phosphate dehydrogenase and the level of ATP synthase alpha subunit during myocardial I/R injury (Cadete et al., 2010). We have previously reported that notoginsenoside NR1 can reduce the energy metabolism disorder caused by I/R by inhibiting the ROCK pathway and increasing the level of mitochondrial ATP synthase subunit, which at least partially protects I/R-injured myocardial tissue (He et al., 2014). Ginsenoside Rb1 can protects against I/R-induced myocardial injury via energy metabolism regulation mediated by RhoA signaling pathway (Cui et al., 2017). The finding of the present study demonstrated that GLXB is able to attenuate the I/R induced activation of RhoA/ROCK, highlighting the participation of this signaling pathway in the action of GLXB.

GLXB is known to contain a range of chemicals including the 10 compounds identified in the present study which belong to nitrogenous compounds and steroidal saponins. Of the 10 compounds, adenosine has been approved by the FDA in 1989 for the treatment of arrhythmias, adenosine, 2,3,4,9-tetrahydro-1H-pyrido [3,4-b] indole-3*R* carboxylic acid and 2,3,4,9-tetrahydro-1H-pyrido [3,4-b] indole-3*S* carboxylic acid have been shown to inhibit human platelet aggregation activity, anti-inflammatory and anti-procoagulant activity (Wang et al., 2016; Yao et al., 2016). However, none of them is known capable of mediating energy metabolism. The component (s) in GLXB responsible for its potential to upregulate ATP 5D and improve energy metabolism in cardiomyocytes remains to be identified by further study.

As a lipid-lowering medication, the potential of simvastatin to protect myocardium from I/R injury also attracts much attention, and anti-apoptosis and anti-inflammation have been proposed as the mechanisms implicated in its action. In line with the previous reports, we observed in the present study that simvastatin is able to lessen the I/R-induced myocardium infarction, attenuate inflammation and anti-apoptosis with efficiency between low and high dose of GLXB tested. Nevertheless, the present study revealed that simvastatin protected energy metabolism disorder in myocardium after I/R challenge in a manner similar to high dose of GLXB. The potential of simvastatin to increase mitochondrial activity and ATP content has been reported in human osteoblasts (Chuang et al., 2013) and by mitochondrial proteomics study of simvastatin effect, but has not been reported in I/R-injured myocardium. The finding of the present study provides a new insight for better understanding the mechanism for the benefit of simvastatin in myocardium undergoing I/R challenge. In addition, the result from simvastatin suggests that the protected effect of GLXB on myocardial I/R injury and energy metabolism disorder found in the present case is partly secondary to its action on plasma lipid, although its direct protection for these insults certainly exists, as demonstrated by the study in normal animals. More study is needed to discriminate the relative contribution of each of the two types of actions to the outcome seen in the present study. Furthermore, the present study shows that in comparison with simvastatin, GLXB is more effective in reducing CK and restoring heart function and cardiac blood flow, though less effective in lowering plasma lipid. Given the well-recognized adverse side effect of simvastatin, GLXB may be used as a potentially alternative for the hyperlipidemia people to prevent myocardial I/R injury.

**Figure 11:**
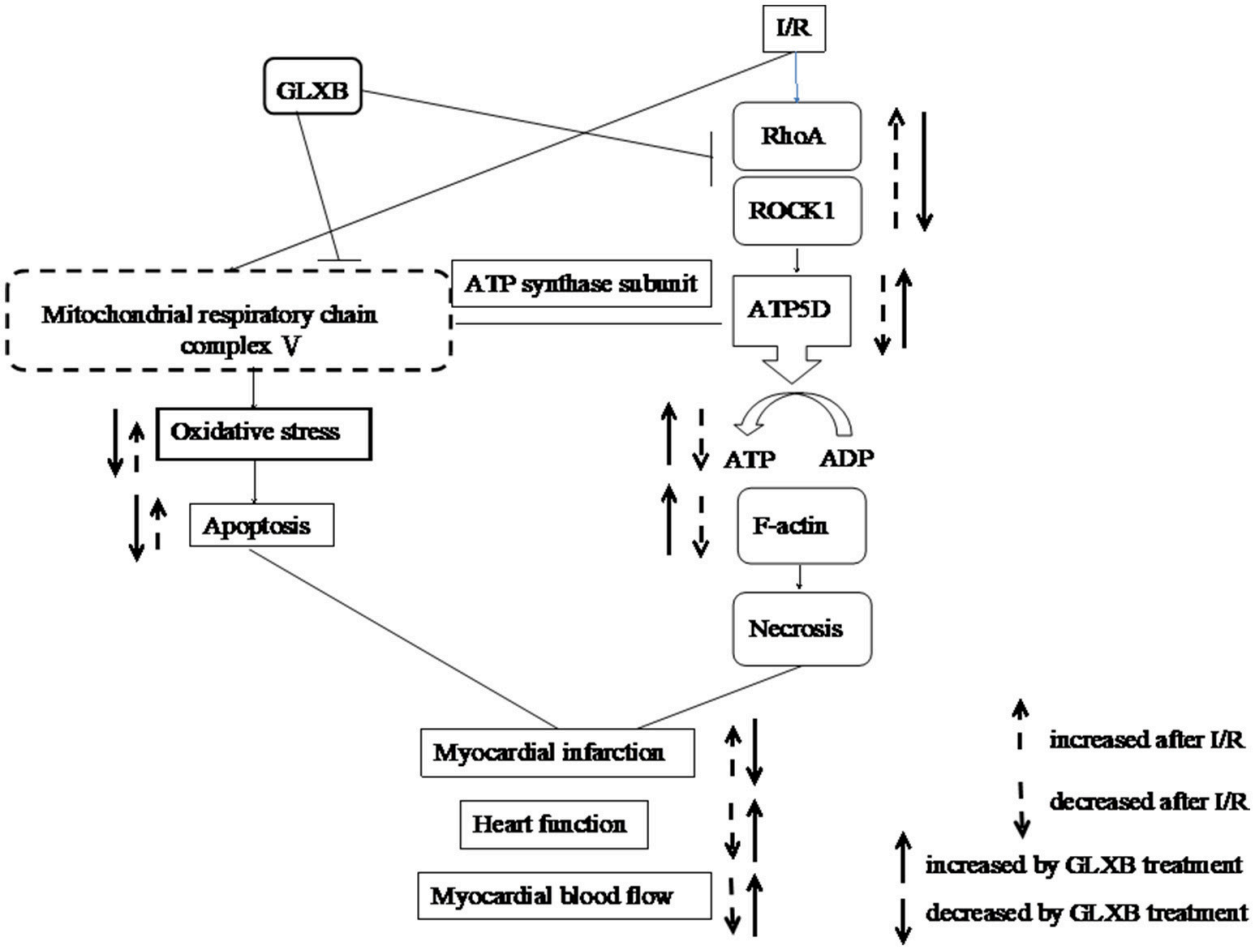
A schematic showing the effect and signaling of GLXB on myocardial I/R injury. I/R activates RhoA/ROCK1 signaling that downregulates the expression of ATP 5D, leading to a decrease of ATP production, disintegrating of F-actin and cardiomyocyte necrosis. which contribute to the decrease in myocardial blood flow, myocardium infarct, and heart dysfunction. In addition, ischemia causes lack of oxygen and glucose which exaggerate the dysfunction of mitochondria respiratory chain, leading to oxidative stress and apoptosis, contributing to myocardium infarct, and heart dysfunction. GLXB inhibits RhoA/ROCK1 signaling, attenuates mitochondria dysfunction, thus improves the outcome of animals exposed to I/R.

In summary, the present study demonstrated that GLXB is able to relieve I/R-induced myocardial injury for the hyperlipidemia subject with an efficiency similar to simvastatin, a potential that is likely related with regulating energy metabolism involving RhoA/ROCK signaling pathway (Figure 11). The result of this study suggests GLXB as an alternative option for the hyperlipidemia patients at high risk of cardiac condition.

## Author contributions

L-LY: Performed the research, analyzed the data, and wrote the manuscript; X-HW, LY, C-SP, and Y-YL: Contributed to animal experiments; YY and W-YZ: Contributed to preparation and composition analysis of GLXB. J-YF, J-YH, and HZ: Revised the manuscript; J-YH, X-SY, and HZ: Designed and funded the research, interpreted the data, and finally approved the submission of this manuscript. All authors have read and agreed with the manuscript.

### Conflict of interest statement

The authors declare that the research was conducted in the absence of any commercial or financial relationships that could be construed as a potential conflict of interest.

## Supplementary material

Figure S1 The full-length representative western blotting bands of ATP5D and GAPDH in different groups as shown with indication of molecular size. The samples derived from the same experiment and gels were processed in parallel. Selected fractions of bands used in the main text were framed as shown in the figure.

Figure S2 The full-length representative western blotting bands of ROCK, RhoA and GAPDH in different groups as shown with indication of molecular size. The samples derived from the same experiment and gels were processed in parallel. Selected fractions of bands used in the main text were framed as shown in the figure.

## Abbreviations

AAR: area at risk
ADP: adenosine diphosphate
AMP: adenosine monophosphate
ATP: adenosine triphosphate
CK: creatinine kinase
CK-MB: creatine kinase isoenzyme MB
CTNT: troponin-T
CTNI: troponin-I
+dp/dtmax: left ventricular maximum upstroke velocity
–dp/dtmax: left ventricular maximum descent velocity
ELISA: enzyme-linked immunosorbent assay
HDL-C: high density lipoprotein cholesterol
HE: hematoxylin-eosin
HFD: high fat diet
HR: heart rate
IA: infarction area
IL-6: interleukin 6
I/R: ischemia/reperfusion
LDH: lactate dehydrogenase
LDL-C: low density lipoprotein cholesterol
LV: left ventricular
LVDP: left ventricular diastolic pressure
LVEDP: left ventricular end diastolic pressure
LVSP: left ventricular systolic pressure
MBF: myocardial blood flow
PBS: phosphate buffered saline
PCI: percutaneous coronary intervention
ROCK: Rho-associated Coiled-coil Containing Protein Kinase
TC: total cholesterol
TG: triglyceride
TNF-α: tumor necrosis factor-alpha
TTC: 2,3,5-triphenyltetrazolium chloride
TUNEL: terminal deoxynucleoitidyl transferase-mediated nick end labeling.
