# Supplementary figures and images for: Gualou Xiebai Decoction, a Traditional Chinese Medicine, Prevents Cardiac Reperfusion Injury of Hyperlipidemia Rat via Energy Modulation
Source: Front Physiol. 2018 Apr 4;9:296. doi: 10.3389/fphys.2018.00296 (PMC5895855; doi:10.3389/fphys.2018.00296)

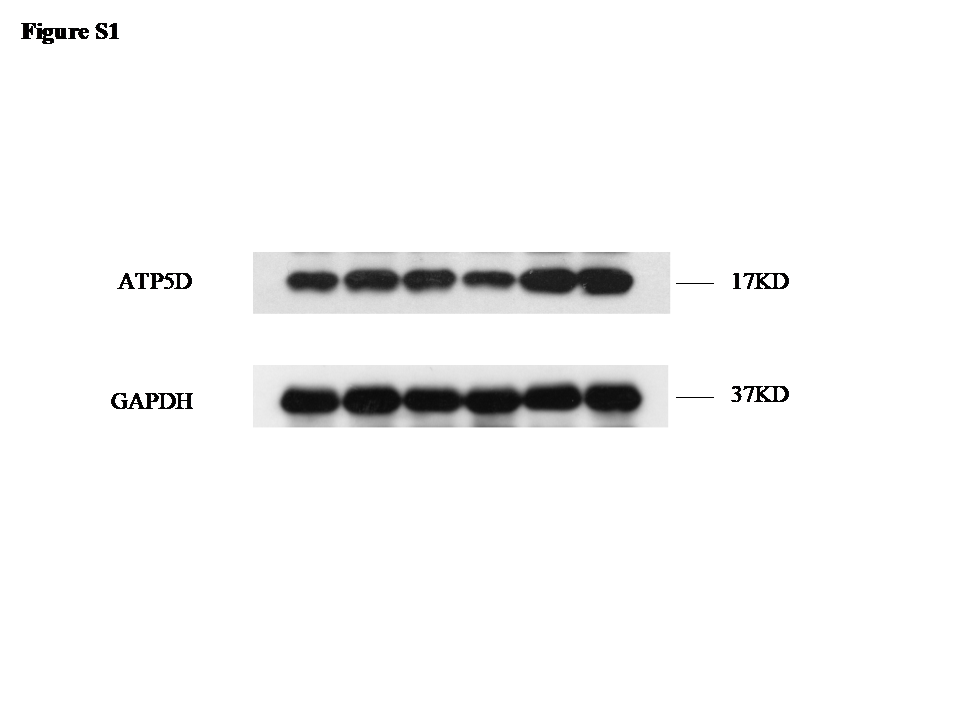

Supplement: Figure S1 — The full-length representative western blotting bands of ATP5D and GAPDH in different groups as shown with indication of molecular size. The samples derived from the same experiment and gels were processed in parallel. Selected fractions of bands used in the main text were framed as shown in the figure. [file Image1.tif]

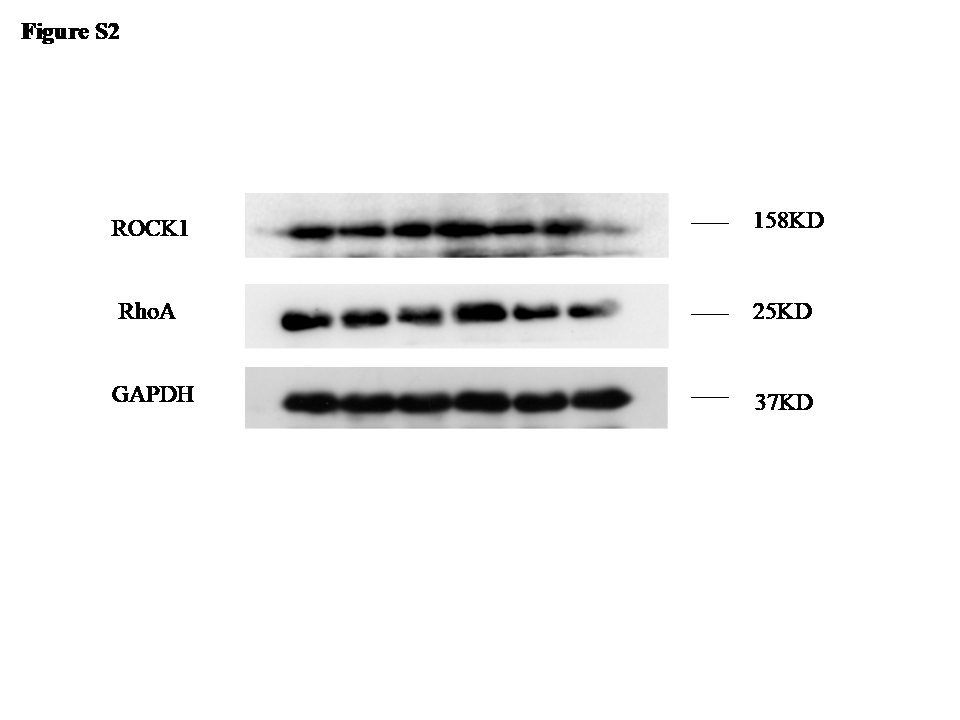

Supplement: Figure S2 — The full-length representative western blotting bands of ROCK, RhoA and GAPDH in different groups as shown with indication of molecular size. The samples derived from the same experiment and gels were processed in parallel. Selected fractions of bands used in the main text were framed as shown in the figure. [file Image2.tif]
